# Chicken Liver from Broilers Fed Wheat Germ Expeller: A Source of Minerals and Energy in the Human Diet

**DOI:** 10.3390/foods14223962

**Published:** 2025-11-19

**Authors:** Zuzanna Goluch, Barbara Król, Gabriela Haraf, Andrzej Okruszek, Kamil Sierżant

**Affiliations:** 1 Department of Food Technology and Nutrition, Wroclaw University of Economics and Business, 53-345 Wroclaw, Poland; gabriela.haraf@ue.wroc.pl (G.H.); andrzej.okruszek@ue.wroc.pl (A.O.); 2 Department of Animal Nutrition and Feed Science, Wroclaw University of Environmental and Life Sciences, 51-631 Wroclaw, Poland; barbara.krol@upwr.edu.pl (B.K.); kamil.sierzant@upwr.edu.pl (K.S.)

**Keywords:** wheat germ expeller, broilers, diet, offal, liver, macroelements, microelements

## Abstract

In recent years, edible by-products (including the liver) have gained growing popularity among consumers. That is why the study aimed to assess the energy value, chemical composition, and mineral content of broiler chicken livers after including wheat germ expeller (WGE) in the feed of the broilers. Liver samples were obtained from 32 Ross-308 chickens (8 individuals per treatment). The control group received a basal diet, whereas the remaining treatments (EX5, EX10, and EX15) were characterized by a partial substitution of ground wheat with 5%, 10%, and 15% WGE. The WGE inclusion did not influence liver weight or chemical composition. However, livers from the CT group showed a higher energy value (*p* ≤ 0.05) than the EX15 group. Sodium and calcium contents were higher in CT and EX5 livers than in EX10 and EX15. No differences were observed in micronutrient levels between groups. A 100 g portion of EX15 livers provided the highest NRV coverage for phosphorus, iron, zinc, and copper, while EX5 livers were richest in calcium and magnesium, and CT livers in manganese. Total Hazard Quotients for Fe, Zn, Cu, and Mn in chicken livers were below 1, suggesting no potential health risk to consumers. These findings indicate that livers, also from WGE-fed broilers, may serve as a valuable dietary source of minerals for people.

## 1. Introduction

The liver of birds, a key component of the digestive system and a major metabolic organ, shares similarities with the mammalian liver in terms of its physiological functions. These functions include the detoxification of substances in feed or metabolites, the secretion of bile, and participation in the metabolism of proteins, carbohydrates, and lipids. The liver also stores specific components, including vitamins (A, D, E, K, and B12), minerals (Fe, Cu, Zn, and Mn), and glycogen. Additionally, it plays a role in hemopoiesis (ectopic erythropoiesis) and serves a protective function. Since the liver receives blood flow from the intestine and general circulation, it can respond to changes in poultry nutrition and environmental conditions. Consequently, the composition of poultry feed influences the blood reaching the liver and, in turn, its physiological functions. This impact can affect the health and welfare of birds [1].

Moreover, post-slaughter poultry liver is considered one of the edible internal organs, along with hearts, gizzards, tongues, pancreases, spleens, brains, intestines, lungs, and kidneys. These organs represent valuable by-products of the meat industry, which can be sold directly to consumers or used as culinary ingredients for producing offal dishes and products, such as pâtés.

For consumers of edible by-products (EBPs), health safety is paramount. Offal products, like meat, are consumed by people of various ages across continents, including Europe, Australia, Africa, Asia, and North and South America. However, consumption patterns depend on factors such as income, customs, traditions, culture, and religion [2]. In Poland, the total consumption of offal in 2024 was estimated at 4.8 kg per capita [3].

Offal products, such as liver, offer an appealing option for consumers due to their low price, high nutritional value, and versatility in culinary preparations, which may include boiling, braising, frying, and grilling. Heat treatment also helps protect against infections caused by Campylobacter bacteria, which are commonly found in poultry liver [4,5]. A recent literature review [6] covering the period from 2014 to 2024 indicated that edible by-products can provide superior amounts of nutrients and bioactive compounds compared to those found in the skeletal muscles of livestock and poultry. Additionally, the optimal use of by-products, including poultry liver, aligns with global trends in sustainability and environmental protection. These trends focus on reducing greenhouse gas emissions, minimizing food waste, and addressing world hunger, especially in developing countries, where offal can help combat malnutrition and enhance food security [7]. The urgency of ensuring food security is growing, as projections from the European Food Safety Authority suggest that the world’s population could rise to 9.8 billion by 2050, resulting in an overall food demand increase in more than 50%, with a nearly 70% rise in the demand for animal-derived foods [8]. Moreover, according to the United Nations’ Sustainable Development Goal (SDG) 12, it is imperative to alter food production and consumption patterns to make them more sustainable, considering the three planetary crises: climate change, biodiversity loss, and pollution [9].

Even though people with food neophobia or food disgust sensitivity might be less willing to consume offal products [10,11], convincing them to do so for dietary or environmental reasons gives hope for popularizing the consumption of these products.

The use of agri-food industry by-products in animal nutrition is a crucial element of sustainable development, offering environmental, economic, and qualitative benefits. Residues such as beet pulp, soybean meal, fruit and vegetable processing waste (e.g., apple, grape, or tomato pomace), and by-products from the oil industry can provide a valuable source of energy, protein, fiber, and bioactive compounds in animal diets. Their use in feed not only reduces food waste and the amount of waste generated but also reduces dependence on conventional feed components and can improve the quality of raw materials and animal products, including offal. Incorporating by-products into the animal feed chain supports the concept of a circular economy. It implements the objectives of the UN Agenda 2030 regarding sustainable agriculture and the efficient use of natural resources [12,13]. These activities are also consistent with the “From Farm to Fork—For a Fair, Healthy, and Environmentally Friendly Food System” strategy [14]. Additionally, using by-products as a source of minerals is more environmentally friendly than mineral supplementation. As demonstrated [15], the use of mineral supplements in the form of synthetic supplements leads to the production of significant amounts of feces, which contribute to eutrophication and soil and water contamination, including the presence of heavy metals. However, it is essential to note that the use of by-products in animal nutrition is associated with certain limitations. The most critical factors include high variability in composition resulting from various processing methods, the need for preservation to stabilize quality, seasonal availability, and a shortened shelf life, especially in the case of raw materials with high moisture and lipid content [12,16]. The qualitative variability of these products can further complicate the proper formulation of feed mixtures [13,17]. The beneficial effects of incorporating agri-food by-products into chicken feed—including, among others, improving slaughter yield, reducing abdominal fat content, and changing the physical, chemical, and sensory properties of meat—are discussed in detail by Sugiharto and Nuengjamnong [13] in their latest, comprehensive literature review.

The European Commission has approved the use of defatted wheat germ expeller (WGE) in animal feed [18], and it is one of the ingredients in feed mixtures for broilers [19,20]. Interest in the application of wheat germ expeller in broiler chicken diets has grown significantly in recent years. Studies indicate that this by-product, when included at varying levels in the feed, can lead to increased feed intake and reduced body weight in chickens, without disrupting the metabolism of proteins, lipids, or carbohydrates [21,22,23,24]. Furthermore, while WGE does not influence the energy content of breast and thigh muscles, it does alter their nutritional composition, potentially helping to meet the Nutrient Reference Values (NRV) for adults.

Among European offal products, poultry liver is the most widely consumed, with an average daily intake of 0.75 g per person. The ability of poultry liver to store various minerals (including heavy metals such as Fe, Zn, Cu, and Mn) is influenced by the composition of the feed given to the birds. Therefore, the nutritional content of this organ as an offal product can significantly impact consumer health and safety. However, it should be emphasized that the content of heavy metals (Fe, Zn, Cu, Mn) in poultry products exceeding FAO/WHO limits may pose a risk to human health after long-term consumption. As demonstrated in recent years, excessive consumption of heme iron (found, among other sources, in the liver of slaughtered animals and poultry) correlates with unfavorable plasma profiles of insulinemia, lipids, inflammation, and type 2 diabetes-related metabolites [25]. An increase of 1 mg of heme iron/day increases the risk of developing type 2 diabetes by 16% [26]. In addition, people with diabetes have an increased risk of developing cardiovascular disease (CVD) [27]. An increased risk of death has also been linked to people with CVD and increased intake of non-heme iron [28]. Increased copper intake may be associated with a higher risk of dementia and a more rapid decline in language function in people with high saturated fat intake [29] and the development of hypertension [30]. In the case of zinc, its adverse effects are primarily associated with supplementation rather than consumption from conventional food sources. Zinc can inhibit copper absorption and thus cause clinical symptoms of copper deficiency (anemia, neurological disorders) [31]. Similarly, excess manganese in the diet, but mainly from drinking water or dietary supplements, can cause neurotoxicity [32]. Therefore, monitoring and controlling metal content in products is essential to minimize health risks for consumers [33,34,35]. There is a lack of data on the nutritional value of chicken livers fed with WGE inclusions. Considering the dietary benefits of WGE and its advantages for poultry breeders—such as sourcing local materials from the oil industry, which is more economical than using foreign components—it is reasonable to pursue further research on how these factors affect the quality of raw materials sourced from broiler chickens.

The study aimed to determine the energy value, proximate composition, and mineral content in the livers of chickens fed diets containing different inclusion levels of WGE as a partial replacement for ground wheat. In addition, THQ was also calculated to assess the potential risk associated with the consumption of heavy metals that come from livers. Furthermore, we sought to estimate how well these minerals meet the Nutrient Reference Values for adults after consuming 100 g of broiler liver. No such research has been conducted so far. In our opinion, this is the first investigation to focus specifically on liver mineral composition, NRV%, and THQ in broilers fed WGE, as some previous studies on WGE have primarily assessed performance, carcass traits, or serum parameters, rather than liver nutrient density. In earlier studies, other authors [36] focused on the impact of using by-products from the agri-food industry on liver enzyme activity, oxidative markers, or gene expression, but not on the mineral content of this organ as a by-product raw material.

## 2. Materials and Methods

### 2.1. Ethics Statement

The animal welfare experiment was authorized by the Advisory Team of the Faculty of Biology and Animal Sciences at the Wrocław University of Environmental and Life Sciences (approval no. 1/2019). Since the study protocol met the necessary criteria, it did not require approval from the Ethics Committee. Throughout the study, the chickens were managed in accordance with European Union regulations and the ethical guidelines established by the Committee [37].

### 2.2. Experimental Design

Livers for analysis were obtained from 32 randomly selected Ross-308 broiler chickens (N = 112) participating in a feeding experiment. One hundred and twelve broiler chickens were randomly assigned to four treatment groups: (1) Control Treatment (CT), which was fed a standard complete diet containing wheat, corn, and soybean meal; and (2) experimental treatments EX5, EX10, and EX15, which were characterized by a partial substitution of wheat meal with 5%, 10%, and 15% WGE, respectively.

Broilers were fasted for 12 h prior to the termination of the experiment, with free access to water. At the conclusion of the trial, eight randomly selected birds from each group were slaughtered in accordance with applicable regulations. The composition of the feeds used in the experiment is presented in Table S1 in the Supplementary Materials. The broiler rearing conditions, feed intake, body weight gains, and pre-slaughter handling of the chicks were detailed in a previous publication [24].

According to the procedure described [38], after slaughter and bleeding of the chickens, their whole livers were dissected, washed under running water, and visible blood, remnants of the bile ducts and gallbladder, external fat, and connective tissue were removed, and then dried with paper towels. Next, the livers were weighed, individually packed into sealed plastic bags, and transported to the laboratory under refrigerated conditions (0–4 °C). The collected material was frozen at −18 °C and kept under these conditions until analysis (about one month). Normalized liver weight was calculated as [39]:

[liver mass (g)/total BW (g)] × 100

### 2.3. Proximate Composition Analysis of Feed and Livers

The proximate composition of feed and liver samples was determined according to the reference procedures described in EN ISO 9831:2004 [40] and the Association of Official Analytical Chemists (AOAC International) [41]. The basic chemical composition of feed and dry matter content in livers was determined on fresh samples. For the remaining analyses, liver samples were lyophilized. Minced and frozen liver samples (−18 °C) were freeze-dried for 48 h under a pressure of 10^−2^ mbar at −55 °C using an Edwards Modulyo freeze dryer (Akribis Scientific Supplies Ltd., Knutsford, UK).

Moisture content was measured according to the AOAC 934.01 method [41]. Approximately 2 g of fresh feed or liver sample was dried in a POL-EKO SLN 115 Eco oven (POL-EKO sp. k., Wodzisław Śląski, Poland) for about 24 h at 105 °C, and the dry matter content was calculated based on the weight loss before and after drying.

Crude ash content was determined according to AOAC 942.05 by incinerating approximately 2 g of fresh feed or lyophilized liver sample in a muffle furnace at 650 °C [41].

The crude protein content was analyzed using the Kjeldahl method (AOAC 984.13) [41]. About 2 g of fresh feed or 0.3 g of lyophilized liver was digested in a FOSS Tecator Digestor for 2 h at 420 °C, and the nitrogen content was quantified using a FOSS Tecator 2300 Kjeltec Analyzer Unit (Foss Analytical, Hillerød, Denmark).

Crude fat was analyzed by the Soxhlet method (AOAC 920.39) [41]. Approximately 2 g of fresh feed or lyophilized liver was extracted with petroleum ether using a BÜCHI Extraction System B-811 (BÜCHI Labortechnik, Flawil, Switzerland).

The gross energy content of both feed and liver samples was determined by calorimetry using a KL-10 automatic bomb calorimeter (PRECYZJA-BIT PPHU Sp. z o.o., Bydgoszcz, Poland).

Crude fiber was determined only in feed samples using the Van Soest method (AOAC 978.10) with filter bags [41]. The sample weight in each bag was approximately 1 g, and the analysis was conducted using an ANKOM 200 Fiber Analyzer (ANKOM Technology, Macedon, NY, USA).

The feed analysis results are presented in the Supplementary Materials in Tables S2 and S3. According to the analysis of the feed used in the experiment, the energy content was very similar in all groups (19.70–19.82 MJ·kg^−1^ DM). The dry matter content increased slightly from CT (90.59%) to EX15 (91.71%). In comparison, crude protein decreased with increasing additive levels (from 23.38% in CT to 22.98% in EX15), resulting in a decrease in the Crude Protein-to-Energy ratio (CP:GE) from 1.19 (CT) to 1.1 (EX15). On the other hand, the Ether extract content increased (from 6.06% in CT to 7.15% in EX15). In the case of crude ash and crude fiber, the average values fluctuate around CA 4.8–5.9% and CF 2.6–3.2%. Nitrogen-free extractives were similar in all groups (52.6–53.3%). It can therefore be concluded that the basic composition of the feed was very similar between groups, with slight differences in fat and protein content.

In the case of macroelements, the content of Pabs (absorbable phosphorus) and calcium was higher in the experimental groups (EX10 and EX15). However, the Na, K, and Mg content in the feed was at a similar level. The content of micronutrients such as iron and manganese showed an upward trend from CT to EX15 (Fe: 173 to 184 mg/kg, Mn: 79 to 95 mg/kg), and zinc (from 74 to 80 mg/kg in EX10). However, the copper content was higher in the control feed (14.4 mg/kg) than in the experimental feeds (11.3–11.8 mg/kg). It can therefore be concluded that the feed additive increased the Fe, Zn, and Mn content, while decreasing the Cu content compared to the control feed.

### 2.4. Analysis of Mineral Content in Feed and Livers

Freeze-dried liver samples were finely ground using a WŻ-1 laboratory grinder (Zakład Badawczy Przemysłu Piekarskiego Sp. z o.o., Bydgoszcz, Poland). For mineral determination, 0.3 g of sample was used for calcium, magnesium, potassium, and sodium analysis, and 1.0 g for copper, manganese, zinc, and iron. The samples were subjected to wet mineralization with a 7 mL mixture of nitric and hydrogen peroxide acids (HNO_3_:H_2_O_2_, 2:3 *v*/*v*) in a MarsXpress microwave digestion system (MARS 6 Microwave Reaction System, CEM Corporation, Matthews, NC, USA). The digestion program consisted of a 10 min heating period up to 190 °C followed by a 7 min hold at 195 °C. After digestion, the samples were transferred to polypropylene tubes and diluted to 50 mL with ultrapure water. Blank digests were prepared under identical conditions.

Macroelements (K, Na, Ca, Mg) and trace elements (Zn, Fe, Mn, Cu) were quantified using flame atomic absorption spectrometry (FAAS, air–acetylene flame) on an AA 240FS SIPS20 spectrometer (Varian, Mulgrave, Australia), in accordance with AOAC [42].

Phosphorus determination was carried out after mineralization with concentrated nitric acid (65%) and perchloric acid using the same microwave digestion system (MARS 6, CEM Corporation, Matthews, NC, USA). Phosphorus content was measured spectrophotometrically by the ammonium vanadomolybdate method at a wavelength of 470 nm, using a HALO DB-20 UV-Vis Double Beam Spectrophotometer (Dynamica Scientific Ltd., Livingston, UK) [22].

Wheat flour certified reference material (SRM 1567b^®^, National Institute of Standards and Technology, Gaithersburg, MD, USA; certificate available at https://tsapps.nist.gov/srmext/certificates/1567b.pdf, accessed on 17 September 2025) was used for quality assurance of mineral determinations in both feed and liver samples. The measured concentrations (mg·kg^−1^) of Ca, P, Mg, K, and Na in SRM 1567b^®^ were 198 ± 20.4, 1198 ± 127, 356 ± 5.3, 1304 ± 158, and 6.74 ± 0.80 (n = 3), respectively, with recovery rates between 89.4% and 103.4% for macroelements. For trace elements (Fe, Zn, Mn, Cu), recovery ranged from 97.0% to 104.2%, confirming the accuracy of the analytical procedure.

### 2.5. Statistical Analysis

The normality of the data distribution was verified using the Shapiro–Wilk test and variance homogeneity using Levene’s test. When necessary, a log transformation was applied to approximate a normal distribution. The statistical analysis included one-way ANOVA. Differences between group means were assessed using Tukey’s post hoc test, with significance levels set at *p* ≤ 0.05 and *p* ≤ 0.01. The tables report mean values along with standard errors of the mean (SEM), based on two parallel measurements. The Principal Component Analysis (PCA) was also carried out to visualize the patterns of association in the data It was applied to the nutritional value of livers (content of moisture, protein, fat, ash, energy, P, Na, Ca, K, Mg, Fe, Zn, Cu, and Mn), consumption of the above-mentioned minerals with feed by chickens, and a feed conversion efficiency value (FCE). All analyses were conducted with Statistica^®^ version 13.1 software. A single bird was the experimental unit in the statistical analysis.

## 3. Results and Discussion

### 3.1. Growth Performance

Tables relating to the chemical composition of the feeds (Tables S2 and S3) and feed intake and weight gain (Table S4) are included in the Supplementary Material. The results regarding the growth performance of broiler chickens in our experiment have been described in detail and published [24]. The study revealed significant differences in feed consumption between the three experimental groups (EX) and the control group (CT). Although birds in the CT group showed lower feed intake relative to body weight (*p* ≤ 0.01) compared to those in the EX5–EX15 groups, they demonstrated the highest body weight gain per 100 g of feed consumed. Adding 5% or 10% wheat germ expeller to the diet increased the intake of gross energy, protein, and fat in the experimental groups compared to CT. The EX10 group showed the highest feed intake and poorest feed efficiency (*p* < 0.01), likely due to an imbalance between crude protein and energy content. In contrast, the CT group had the lowest feed and protein intake relative to final body weight (*p* < 0.01). Lower feed consumption in the CT may be linked to a reduced intake of minerals, such as P, Na, Ca, Mg, Fe, Zn, and Mn, compared to the other groups. The EX10 chickens had the highest intakes of P, Na, Ca, and Zn; EX5 had the highest intakes of K and Mg; and EX15 showed the highest intakes of Fe and Mn, reflecting the mineral composition of their diets. No differences were observed in Cu intake among groups.

### 3.2. Weight and Proximate Composition of Livers

In general, the liver weight of chickens varies with age and body weight [39]. The study found no significant differences in liver weight between the groups of chickens when expressed as grams per 100 g of body weight (BW) (Table 1). However, the average liver weight of the Ross-308 chickens tested at 43 days was higher than the findings of other researchers for the same breed at 35 days (40.11 g), 42 days (43.6 g), and 45 days (41.50 g) [43,44,45]. Normalized liver weight also did not differ significantly between the chicken groups (1.92–2.07%) and was similar to that reported in the literature for 35-day [39,46] and 42-day [45,47] broilers. However, it was lower than in Coob 500 broiler chickens from the control group or after the use of Fermented Wheat Germ Extract (FWGE) at 0.1 and 0.3% per kg of feed after 45 days of rearing (respectively: 2.52; 2.56, 2.62%) [23].

The liver is a key metabolic organ that regulates the body’s energy metabolism through multiple nutritional, hormonal, and neuronal signals, which control the liver’s metabolism of glucose, lipids, and amino acids [48]. In chicken nutrition, feeds balanced in energy and protein content have been shown to optimize liver function [49]. In our study, the energy value of chicken livers from all groups was higher than reported in the literature (0.80–0.87 MJ/100 g vs. 0.385–0.67 MJ/100 g wet weight basis—WWB). Information on product labels is also provided to consumers in kcal [46,50,51,52,53,54,55]. The above-mentioned energy value of the tested livers, converted into kcal, ranges from 191.08 to 207.8 kcal, compared to 91.96 to 160.03 kcal, respectively. Despite the higher energy content of the EX15 group’s feed (Table S3) and no difference in energy intake compared to the CT group (Table S4), livers from EX15 chickens had significantly (*p* ≤ 0.05) lower energy values. A trend towards lower liver weight was observed in the EX10 and EX15 groups, accompanied by a concomitant tendency towards lower fat content in the EX15 group. The lowest (*p* = 0.051) fat content in the EX15 livers translated into significantly lower energy value (by approximately 10%) compared to the CT livers.

In our study, the use of dietary WGE in the feed did not affect the moisture content of chicken liver, although it was lower than reported in the literature (70–77% WWB) [46,50,51,52,53,54,55].

According to the literature, the fat content of broiler chicken livers ranges from 4% to 7% WWB [46,50,51,52,53,54,55], which is consistent with our findings (Table 1). However, there were no significant differences in the fat content of this organ between the chicken groups, even though it was observed to be significantly (*p* ≤ 0.01; *p* ≤ 0.05) more consumed with the feed of the EX5-EX15 groups (Table S4). Furthermore, during the preparation of the livers, no changes in their color were observed, which could occur with an increase in fat content. As we showed in the same experiment [22], adding WGE to chicken feed did not induce adverse changes in chickens’ carbohydrate and lipid metabolism.

The protein content of fresh broiler chicken livers ranges from 16 to 20% [46,50,51,52,53,54,55]. In our study, the protein content of chickens’ livers did not differ between groups despite the significantly (*p* ≤ 0.05; *p* ≤ 0.01) higher intake of this feed component in groups EX5-EX15 compared to CT (Table S4). The protein content in the liver depends on protein metabolism in the chicken’s body. Regarding protein metabolism, in the same experiment [22], only the β2-globulin fraction in the blood serum differed significantly between EX5–EX15 and the control group, suggesting the need for further research.

In our study, the ash content of chicken livers was not affected by the inclusion of WGE in the diet and remained within the range of 1.0–1.43% WWB, as reported in the literature [46,50,51,52,53,54,55].

### 3.3. Macroelements

Phosphorus is among the minerals found mainly in bones and blood plasma (in organic and inorganic compounds) and in extracellular and intracellular spaces. It is an essential component of high-energy compounds such as ATP, ADP, and phosphocreatine. Combined with proteins and lipids, it forms phospho-proteins and phospholipids, the building blocks of cell membranes and components of nucleic acids and nucleotides. As part of the blood’s buffer compounds, it regulates the body’s acid-base balance [56]. According to literature data, the mineral content of chicken livers varies depending on breed, diet, and rearing conditions. Across all treatment groups, the levels of this element in chicken livers did not differ significantly (Table 2), despite differences in the content in the feed (Table S3) and a significant difference in the amount of intake of this element with the feed (Table S4). The phosphorus content found in the livers of the CT groups is similar to that described in the literature, ranging from 258.33 to 320.0 mg/100 g WWB [46,50,51,52,53,54,55]. In contrast, in the experimental groups EX10 and EX15, it was slightly higher.

As sodium cannot be produced endogenously, it is considered an essential mineral that must be provided in the diet. It is the main cation in extracellular fluid, and a small amount is also in intracellular fluid [57]. It is involved in maintaining electrolyte and water balance (hydromineral regulation). The study found an effect of WGE application on sodium content in chicken livers (Table 2). Despite the low intake of this element with feed in the CT and EX5 groups (Table S4), the sodium content was significantly (*p* ≤ 0.01) higher in the livers of chickens from these groups compared to the EX10 and EX15. A reduced sodium content in feed may contribute to increased feed intake (a compensatory effect), osmoregulation, and storage of this element in tissues, including the liver. The liver is not the primary site of sodium deposition. We believe that higher amounts of this element were deposited in the breast muscles of EX15 chickens compared to CT, as we have previously demonstrated [24]. The sodium content found in the livers of chickens from CT groups and experimental groups was higher than that reported in the literature for broiler chickens (range of 67–85 mg/100 g WWB) [46,50,51,52,53,54,55].

Despite significantly lower Ca intake by CT and EX5 chickens compared to the other experimental groups (Table S4), their livers had significantly higher Ca content (Table 2) compared to EX15 (*p* ≤ 0.05). According to literature data, chicken liver (including Ross-308) contains small amounts of calcium, ranging from 7 to 15 mg/100 g WBB [46,50,51,52,53,54,55,58,59]. It is because it is mainly found in blood plasma (in the form bound to plasma proteins with albumin and globulins, and in the form bound to other ions: citrate, phosphate, sulfate, or lactate), in bones (in the form of hydroxyapatite, carbonate, and phosphate), and in the nervous and muscular systems [60]. The Ca content found in the livers of chickens from CT and EX5 was higher, and that of chickens from EX10 and EX15 was similar to that presented above from the literature.

Potassium is the primary intracellular ion that regulates water and electrolyte balance. Along with sodium, it also plays a role in the body’s acid-base balance [61]. According to the literature, the potassium content of chicken livers ranges from 215 to 300 mg/100 g WWB [46,50,51,52,53,54,55], but in our experiment, it was lower. However, WGE had no significant effect on the liver content of this element (Table 2) compared to CT. It may be because the potassium content of the feed was similar (Table S3), although it was found to be significantly (*p* ≤ 0.05) more consumed by chickens in the EX5 group compared to CT (Table S4).

Magnesium is predominantly found in the body, with approximately 99% of it in the intracellular space. Most magnesium (50–65%) is found in the bones, which work in conjunction with calcium and phosphorus to help build the skeleton. The remaining magnesium is distributed in muscles, soft tissues, and organs (34–39%), with only 1–2% present in the blood and extracellular fluids. Magnesium is crucial in almost every primary metabolic and biochemical process within cells. It is essential for various bodily functions, including bone development, neuromuscular function, signaling pathways, energy storage and transfer, and the metabolism of glucose, lipids, and proteins. Additionally, magnesium contributes to the stability of DNA and RNA and is essential for cell proliferation [62]. Higher (*p* ≤ 0.05) Mg amount (Table 2) was determined in the livers of chickens from the EX5 group, compared to the CT group, even though all experimental groups (EX5-EX15) had higher (*p* ≤ 0.01) intakes of this element compared to the CT group (Table S4). It confirms the relationship described in the literature [63] that higher Mg content in the feed of the EX5 group and its higher consumption resulted in greater accumulation of this element in the chicken liver. Furthermore, magnesium is less susceptible to binding by phytates present in the feed than zinc, which may lead to its better bioavailability [64].

According to the literature, the magnesium content of chicken livers (including Ross-308) ranges from 19 to 32 mg/100 g WWB [46,50,51,52,53,54,55,58,59], but in our study, the determined content in the EX5 group was higher.

Since no studies were found to determine the content of macronutrients in the livers of chickens fed WGE, the discussion in this area is limited, and our research is a novelty. Comparing our results to those contained in food databases is possible. However, as other authors have noted [6], the information contained therein does not reveal how it was obtained.

### 3.4. Microelements

Copper is essential for cellular respiration, defense against free radicals, neurotransmitter function, and tissue biosynthesis. It influences enzyme activity as a cofactor or a basic structure of many metalloenzymes, such as superoxide dismutase, ceruloplasmin, lysyl oxidase, cytochrome oxidase, and tyrosinase. It is also essential for the proper development of antibodies and white blood cells, as well as the production of antioxidant enzymes [65]. In our study, the lack of significant differences in copper intake between the groups of chickens (Table S4) resulted in similar copper contents in their livers (Table 3). According to the literature, the Cu content of chicken livers ranges from 0.3 to 0.51 mg/100 g WWB [46,50,51,52,53,54,55], and the one we found was higher in all groups.

Iron plays an essential role in oxygen transport, nucleic acid replication and repair, host defense, cellular proliferation, and oxidative metabolism, and is required for adequate erythropoietic function and cellular immune response. Approximately 75% of the iron in the body is bound to erythrocytes as hemoglobin (Hb), while the remaining amount is in the form of free iron, which has cytotoxic potential and can act as a catalyst in the production of reactive oxygen species (ROS), contributing to metabolic disorders [66]. The liver is the primary site in the body for storing iron [46]. In our experiment, although significant differences were found in the intake of this element with feed between the groups of chickens studied (Table S4), no significant changes were observed in its content in livers (Table 3). The liver iron contents determined in the present study were higher than those reported in the literature (6.7–12.93 mg/100 g WWB) [46,50,51,52,53,54,55]. In our opinion, the higher (though not statistically significant) iron content in the livers of chickens from the EX10-EX15 groups could have resulted, firstly, from a higher (by 4.2–6.30%) content of this element in the feed (Table S3) and its higher consumption (Table S4) compared to the control group. Secondly, the higher Fe accumulation in the livers of chickens from the EX10-EX15 groups may also result from antagonism towards copper [67]. The higher Fe content in the feed of these chicken groups (with a simultaneous lower Cu content and lower consumption, Tables S3 and S4) could have inhibited Cu absorption in the intestines, as these metals compete for common transport proteins (DMT1—divalent metal transporter 1), which results in a decrease in Cu in the bloodstream [68]. Copper, among others, is essential for the proper functioning of ceruloplasmin, which oxidizes Fe^2+^ → Fe^3+^, enabling its binding to transferrin and transport. When blood copper concentration decreases, iron accumulation in the liver can occur, accompanied by a concomitant reduction in copper in peripheral tissues. In this experiment, the higher Fe content in the livers of EX10 chickens was accompanied by lower Cu content in the livers of EX15 chickens. However, despite the higher Fe content, higher copper content was also observed in the livers of EX15 chickens. Therefore, in our opinion, explaining this fact (although not statistically significant) would require blood tests of the chickens’ metabolism of both elements. An evident relationship between these two metals is visible in the CT group, where higher Cu content in the feed, higher intake, and higher content in the liver were associated with lower Fe content in the feed and lower intake and content in the chicken liver. Other authors demonstrated a significant (*p* ≤ 0.05) effect of copper content in the feed on lower iron content in the liver [69]. We indicated increased Fe metabolism in the livers of EC10-EX15 chickens in our earlier work [16], in which we observed an increase in the β2-globulin fraction (containing transferrin, ferritin, and hemopexin) in the chickens’ blood.

After iron, zinc is the body’s second most abundant micronutrient. Zn is 70% bound to albumin in the bloodstream, but most of the body’s zinc is found in skeletal muscles and bones. It is involved in numerous cellular processes, including protein synthesis, nucleic acid metabolism, gene transcription, cell proliferation and differentiation, and mitosis. In addition, it is involved in collagen matrix synthesis, mineralization and bone turnover, regulates intracellular signaling pathways of innate and adaptive immune cells, influences immune responses (antibody production, inflammatory signaling and lymphocyte differentiation), is essential for the formation and structural stability of insulin, for the proper functioning of the central nervous system and male fertility. In chickens, zinc is absorbed into enterocytes primarily in the upper small intestine via ZIP (SLC39) and ZnT (SLC30) transporters, where it is bound by metallothionein (MT), which regulates its intracellular pool and growth/retention [70]. The chemical form of this element present in the feed influences its absorption. Organic forms (chelates, amino acid complexes) exhibit higher bioavailability than inorganic salts (e.g., ZnO, ZnSO_4_) because they can utilize peptide/amino acid transport and/or are less likely to be bound by inhibitors (such as phytates) in the intestinal lumen. Therefore, phytinase is added to chicken feed. The bioavailability of zinc from feed is also influenced by competition with other ions, such as Cu, Fe, and Mn, due to the same loading, transporters (DMT1, divalent metal transporter 1, ZIP, and ZnT), and binding sites in proteins [71]. However, it cannot be stored in larger quantities in the body [72]. Although this was significant, the lowest Zn intake (*p* ≤ 0.01) was observed in the CT group chickens (Table S4). In the study conducted, the zinc content of chicken livers did not differ significantly between groups. It confirms the body’s ability to maintain homeostasis in the body, because despite the higher content of Fe and Mn in the feed of EX10-EX15 chickens (Table S3) and significantly higher consumption of these elements (Table S4), the expected significant reduction in Zn content in the livers was not observed, which would result from antagonism between these components. The zinc contents determined in the present study in groups CT and EX5 were lower than those reported in the literature (2.69–5.99 mg/100 g WWB) [45,50,51,52,54,55], and similar in the experimental groups.

Manganese is a component of metalloenzymes (arginase, glutamine synthetase, and pyruvate carboxylase), activates manganese superoxide dismutase (MnSOD), and is an activator of a large number of hydrolases, kinases, decarboxylases, and transferases. Manganese participates in metabolizing amino acids, lipids, and carbohydrates and the synthesis of proteoglycans in bone formation [73,74]. In our study, significant differences (*p* ≤ 0.01) in manganese intake were observed (Table S4), which was influenced by the WGE in the feed; however, this did not translate into differences in Mn content in chicken livers (Table 3). A trend (*p* = 0.087) suggests that with increasing WGE in the feed, Mn intake rises; however, there is a trend towards a decrease in its content in the livers. According to the literature, the Mn content of chicken livers ranges from 0.255 to 0.330 mg/100 g WWB [38,43,44,47,48], whereas the one we found was higher in all groups.

In summary, in our view the total crude ash content (Table S2) as well as the mineral composition of the EX10 and EX15 diets indicate that the feed was directly enriched with these elements through the addition of WGE, since the 0.25% premix supplementation was identical across all groups. However, changes observed in the chickens’ livers—namely a reduction in sodium content and an increase in calcium and magnesium levels (in the EX5 group)—suggest an additional, indirect effect related to the metabolism of these elements in the body to maintain homeostasis. Although several studies have shown [75,76,77,78] that raw or fermented plant by-products (such as grape pomace, blueberry pomace, pineapple pomace, and rapeseed cake) can affect the ash and mineral content of diets due to their own content of macro- and microelements (K, P, Ca, Fe, Zn, Cu, Mn), their influence on the mineral composition of broiler chicken livers has not been investigated.

### 3.5. Risk Assessment Calculation

A Risk Assessment Calculation was performed for Fe, Zn, Cu, and Mn. The Estimated Daily Intake (EDI) for each microelement was calculated using the following equation:

$$EDI=\frac{C\cdot IR}{BW}$$

where C—concentration of target element in food (mg/kg), IR—intake rate (kg/day), and BW—body weight (kg).

To calculate the EDI value, it was assumed that the consumption of liver in chicken in Europe is 0.27 kg/year/person (~0.00074 kg/day), and the average body weight of an adult is 70 kg [6]. Next, the Hazard Quotient (HQ) for each element was calculated using [79]:

$$HQ=\frac{EDI}{RfD}$$

where EDI—Estimated Daily Intake, RfD—Reference Dose = 0.7 mg/kg BW/day for Fe, 0.3 mg/kg BW/day for Zn, 0.04 mg/kg BW/day for Cu, and 0.1 mg/kg BW/day for Mn [80,81,82,83].

The Total Hazard Quotient (*THQ* $=\;{HQ}_{Fe}+\;{HQ}_{Zn}+{HQ}_{Cu}+{HQ}_{Mn}$), summarizing the combined exposure to all four elements was:

$${THQ}_{CT}=\;0.00322+0.00091+\;0.00214+\;0.00049\approx 0.00675\;{THQ}_{EX5}\;=\;0.00328+0.00091+\;0.00201+0.00049\approx 0.00688\;{THQ}_{EX10}\;=\;0.00384+0.00096+\;0.00193+0.00042\approx 0.00715\;{THQ}_{EX15}\;=\;0.00402+0.00106+\;0.00227+0.00041\approx 0.00776$$

Because the THQ values were below 1, the cumulative intake of Fe, Zn, Cu, and Mn from chicken liver can be considered safe for consumers. Also, no studies on the content of microelements in the livers of chickens fed WGE were found; therefore, the discussion in this area is limited, and our study is a novelty in this field. However, we can compare the content of these elements in the livers of other animals. According to data from food composition databases [50,51], the content of Fe, Zn, Cu, and Mn in raw pork liver is 233.0; 57.7; 6.8; 3.4 mg/kg, in beef: 40.9; 40.0; 97.6; 3.1 mg/kg, in veal: 0.79; 0.84; 0.55; 2.8 mg/kg, in turkey: 89.4; 33.7; 8.63; 2.90 mg/kg, and in duck: 305.3; 30.7; 59.6; 2.58 mg/kg. Assuming the above-mentioned content and consumption levels of chicken liver (~0.00074 kg/day) by an adult with an average body weight of 70 kg, the THQs will be 0.008 for pork liver, beef liver 0.028, veal liver 0.0190, turkey liver 0.005, and duck liver 0.022. All these values are well below 1, which means that the consumption of these animal livers (at a rate of 0.27 kg/year) does not pose a toxicological risk due to these elements. However, the consumption [6] of pork, beef, and veal liver is lower than that of chicken liver (0.55, 0.36, and 0.27 g/day, respectively), and the THQs calculated for these elements are 0.00375, 0.02063, and 0.00693, respectively. Hence, they pose even less of a toxicological risk to consumers.

### 3.6. Principal Components Analysis

The purpose of the PCA was to determine which treatment had the most significant influence on the liver’s chemical composition and mineral content. The first two Principal Components (PCs) accounted for 50% of the total variance. The PC1 explained 36% of the variance and had strong positive loadings (absolute value equal to or higher than 0.58) for P, Na, Ca, Mg, Fe, Zn, Mn, and K intake by the chickens, whereas it was negatively correlated with FCE and Na liver content (Table 4). Accounting for 14% of the overall variance, PC2 is positively linked to Mg concentration in the liver and inversely related to K content. Table 4 also shows that PC1 is highly negatively correlated with the CT group and PC2 is strongly positively correlated with the EX5 group.

**Figure 1:**
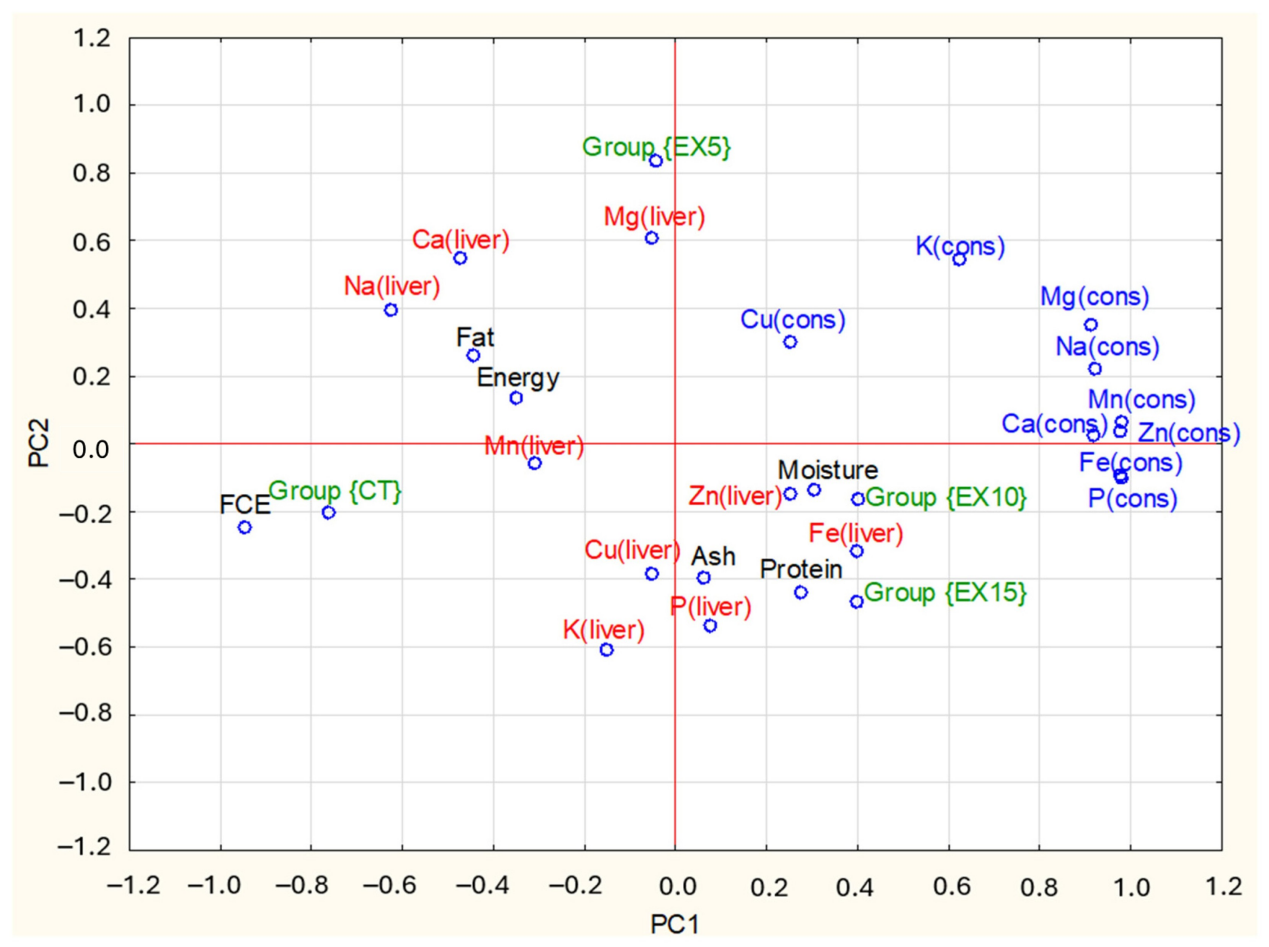
Loadings plot of the two first PCs (variable designations as in Table 4).

The relationship between the study results and PCs is shown graphically in Figure 1. The PC1 and PC2 axes represent the first and second principal components. Their values are a measure of variability in the data—the farther from the center, the greater the influence of a given variable on the differences between the study groups. Values close to each other indicate a similarity of characteristics. In Figure 1, the points corresponding to the experimental and control groups are far apart, indicating a significant effect of the WGE feed component on the nutritional value of Ross 308 chicken livers. In the exact figure, the EX10 and EX15 groups are located close to each other, which is why they are similar in terms of the analyzed liver characteristics but different from the control group and EX5. The PCA also shows that the higher consumption of minerals such as K, Na, Mn, Ca, and Cu by chickens does not translate into a higher content of these components in the obtained livers. A clear positive correlation exists between consumption and content in the liver for Zn and Fe. It can, therefore, be concluded that the consequence of a higher proportion of wheat germ expeller in the feed (10 and 15%) is a higher consumption of Zn and Fe by chickens and, as a result, a higher content of these minerals in the chicken livers. The obtained results of the analysis also confirm that the control group differs significantly from the others in terms of the FCE (Feed Conversion Efficiency) index value and strongly positively correlates with its value. It means that adding wheat germ expeller did not have a positive effect on this production index.

### 3.7. Broiler Liver and Nutrient Reference Values

The NRVs are established daily nutrient intake levels based on current scientific knowledge for maintaining good health. These values are primarily used in food labeling to inform consumers about the nutrient content and support them in making informed dietary decisions. By indicating the percentage of NRVs on packaging, manufacturers provide consumers with a simple way to assess how a specific portion of a product contributes to their daily nutritional requirements. It fosters a deeper understanding of the relative nutritional value of various foods and encourages healthier purchasing decisions. NRVs can also help consumers meet recommended intakes or avoid excessive consumption. Beyond consumer guidance, NRVs are also used by food manufacturers in product design and labeling, as well as by food safety managers, to establish reference values on labels. Nutritionists and health care professionals use them to plan menus and assess patients’ nutritional status. NRVs are also used to monitor nutrition programs, assess food availability, and support educational and development activities related to healthy eating. The Codex Committee on Nutrition and Foods for Special Dietary Uses [84] has also defined NRVs—reference levels for mineral requirements (Table 5).

Based on calculations, 100 g of chicken liver from the EX15 group provides the highest percentage coverage of the NRVs for adults regarding P, Fe, Zn, and Cu. In contrast, the same portion of chicken liver from the EX5 group offers the greatest coverage for Ca and Mg, while the CT group primarily meets the NRVs for Mn (see Table 5). However, only P, Fe, Cu, and Zn are present in livers in significant amounts. In this context, a considerable amount of a mineral in a food item is defined as 15% of the reference intake values, based on a 100 mL or 100 g quantity for products other than beverages [85]. Chicken liver from any group will not be a significant food source for Ca, Mg, and Mn in the human diet.

Chicken liver from EX15 can be an important dietary source of phosphorus, but higher intake does not necessarily lead to adverse health effects such as hyperphosphatemia. This condition is primarily linked to the excessive use of phosphates as preservatives in food and beverages [86]. Due to their significant Fe, Zn, and Cu content, chicken livers from all the groups studied can be a good source of these elements in the human diet, especially in cases of deficiencies or as an occasional addition to the daily menu. However, the serving size of such liver should be individually tailored to the consumer’s needs to avoid excessive consumption of these metals. It is also worth considering that heat treatment (boiling, frying, braising, baking) and the equipment used can affect the finished product’s nutrient content, including minerals [87].

Poultry producers must consider whether the costs incurred in producing chickens with WGE will be offset by revenues from selling raw materials, including livers. Consumer acceptance and positive evaluation of the organoleptic qualities of these products may be crucial in this assessment. It may be the case that, despite higher production costs, a group of consumers will be willing to pay a premium for products with verified health-promoting properties derived from sustainable and welfare-oriented farming systems. In this context, informing consumers about the health-promoting qualities of such products on the label can effectively increase their market appeal and demand [88,89].

## 4. Conclusions

Livers from all experimental groups contained both macroelements (in the order: P > K > Na > Mg > Ca) and microelements (Fe > Zn > Cu > Mn). Among the analyzed samples, a 100 g portion of liver from the EX15 group provided the highest percentage of the Nutrient Reference Values (NRVs) for phosphorus, iron, zinc, and copper. In contrast, livers from the EX5 group showed the greatest NRV coverage for calcium and magnesium. These findings suggest that broiler livers, particularly those from chickens fed WGE, can be considered an important source of essential minerals. Highlighting this information on product labels could help consumers make more informed dietary choices and promote a more diversified and balanced diet. Moreover, the enhanced nutritional quality of livers obtained from birds fed with WGE may be of interest to poultry meat processors. Notably, the calculated Total Hazard Quotients (THQs) for Fe, Zn, Cu, and Mn were all below 1, confirming that consumption of chicken liver does not pose a health risk to humans. One limitation of our study is the absence of an economic analysis comparing the use of wheat germ expeller (WGE) to ground wheat in broiler chicken feed. However, it is essential to emphasize that the overall cost-effectiveness depends on several factors: the market price of WGE relative to the ingredient it replaces (in this case, ground wheat), its availability, and the slight reduction in final body weight observed in broiler chickens. Given the current volatility in feed ingredient prices, a broader economic evaluation is not feasible at this time. However, the inclusion of WGE in chicken feed aligns with the global trend toward more sustainable production practices. Another limitation of this study is that it did not include physiological assessments to explain the mechanisms regulating mineral balance in chickens. Therefore, future research should focus on evaluating whether experimental diets containing WGE—characterized by higher levels of Ca, Mg, Fe, Zn, and Mn and lower levels of Na, K, and Cu compared to standard feed—affect systemic homeostasis.

## Figures and Tables

**Table 1:** Weight and proximate composition of broiler liver.

Trait	CT	EX5	EX10	EX15	SEM	*p* Value
Liver weight (g)	50.9	49.3	45.8	43.3	1.05	0.060
Normalized liver mass (%) (g/100 g of BW)	2.03	2.07	2.02	1.92	0.03	0.437
Gross energy (MJ/100 g WWB)	0.87 ^a^	0.85	0.85	0.80 ^b^	0.090	0.028
Moisture (% WWB)	68.4	68.4	68.9	70.3	0.354	0.193
Crude fat (% WWB)	5.86	5.66	5.00	4.09	0.266	0.051
Crude protein (% WWB)	20.2	19.5	20.4	20.5	0.196	0.291
Crude ash (% WWB)	1.46	1.40	1.45	1.47	0.019	0.596

WWB—wet weight basis; further explanations are given in Section 2; significance: ^a,b^ *p* ≤ 0.05.

**Table 2:** Macroelements composition (mg/100 g WWB) of broiler liver.

Minerals	CT	EX5	EX10	EX15	SEM	*p* Value
P	394.0	344.7	413.0	419.4	12.3	0.262
Na	139.2 ^A^	123.9 ^A^	101.3 ^B^	93.5 ^B^	4.42	0.001
Ca	19.4 ^a^	24.2 ^a^	12.3	11.1 ^b^	1.67	0.010
K	218.6	187.0	210.5	204.2	5.22	0.205
Mg	29.4 ^b^	35.9 ^a^	30.7	30.4	0.79	0.013

Explanations are given in Section 2; Significance: ^A,B^ *p* ≤ 0.01; ^a,b^ *p* ≤ 0.05.

**Table 3:** Microelements composition (mg/100 g WWB) of the broiler liver.

Minerals	CT	EX5	EX10	EX15	SEM	*p* Value
Cu	0.81	0.76	0.73	0.86	0.020	0.114
Fe	21.3	21.7	25.4	26.6	0.981	0.218
Zn	2.58	2.58	2.72	3.01	0.080	0.139
Mn	0.46	0.43	0.40	0.39	0.010	0.087

Explanations are given in Section 2.

**Table 4:** Loadings for the two first PCs.

	Items	PC1 ^1^	PC2 ^1^
Mineral contents of livers:	P (liver)	0.08	−0.54
	Na (liver)	−0.62	0.40
	Ca (liver)	−0.47	0.55
	K (liver)	−0.15	−0.61
	Mg (liver)	−0.05	0.61
	Fe (liver)	0.40	−0.32
	Zn (liver)	0.25	−0.15
	Cu (liver)	−0.05	−0.39
	Mn (liver)	−0.31	−0.06
Chemical composition of livers:	Moisture	0.31	−0.14
	Protein	0.28	−0.44
	Fat	−0.44	0.26
	Ash	0.06	−0.40
	Energy	−0.35	0.13
Mineral consumption with feed:	P (cons)	0.98	−0.10
	Na (cons)	0.92	0.22
	Ca (cons)	0.92	0.02
	K (cons)	0.63	0.54
	Mg (cons)	0.91	0.35
	Fe (cons)	0.98	−0.10
	Zn (cons)	0.98	0.04
	Cu (cons)	0.25	0.30
	Mn (cons)	0.98	0.06
	FCE ^2^	−0.95	−0.25
Groups of chickens ^3^:	Group {CT}	−0.76	−0.20
	Group {EX5}	−0.04	0.83
	Group {EX10}	0.40	−0.16
	Group {EX15}	0.40	−0.47

^1^ PC1, PC2—first and second Principal Components; ^2^ FCE—Feed Conversion Efficiency; ^3^ Explanations are given in Section 2.

**Table 5:** Nutrient Reference Values (NRVs, mg/100 g) for adults and the proportion (%) provided by the liver of broilers.

Mineral	NRVs ^1^	CT	EX5	EX10	EX15
P	700	53.3	49.21	59.0	59.9
Ca	1000	1.94	2.42	1.21	1.11
Mg	310	9.5	11.6	9.9	9.8
Fe	14	152.1	155.0	181.4	190.0
Zn	11	23.5	23.5	24.7	27.4
Cu	0.9	90.0	84.4	81.1	95.6
Mn	3	15.3	14.3	13.3	13.0

^1^ NRVs—Nutrient Reference Values; further explanations are given in Section 2.

## Data Availability

The original contributions presented in this study are included in the article/Supplementary Materials. Further inquiries can be directed to the corresponding author.

## Supplementary Materials

The supporting information can be downloaded at: https://www.mdpi.com/article/10.3390/foods14223962/s1. Table S1. Dietary ingredients content, metabolizable energy (ME) value (MJ), and essential nutrients of experimental diets (g/kg of feed). Table S2. Chemical composition of wheat germ expeller (Mean, SD). Table S3. Chemical composition of the experimental diets (applies to the finisher’s diet). Table S4. Feed intake and weight gain of male 43-day-old Ross-308 broilers (MEAN, SEM, *n* = 32).

## Abbreviations

The following abbreviations are used in this manuscript:

AOAC	Association of Official Analysis Chemists
BW	Body Weight
CT	Control Treatment
EBP	Edible By-Products
EDI	Estimated Daily Intake
EX	Experimental diet
FAO	Food and Agriculture Organization of the United Nations
FAAS	Flame Atomic Absorption Spectrometry
FWGE	Fermented Wheat Germ Extract
HQ	Hazard Quotient
NRV	NRV
PCA	Principal Component Analysis
RfD	Reference Dose
THQ	Total Hazard Quotient
USDA	United States Department of Agriculture
WEG	Wheat Germ Expeller
WHO	World Health Organization
WWB	Wet Weight Basis
