# Supplementary material for: Chicken Liver from Broilers Fed Wheat Germ Expeller: A Source of Minerals and Energy in the Human Diet
Source: Foods. 2025 Nov 19;14(22):3962. doi: 10.3390/foods14223962 (PMC12651864; doi:10.3390/foods14223962)
Supplement: Supplementary file 1 [file foods-14-03962-s001.zip › foods-3964747-supplementary.pdf]

Table S1. Dietary ingredients content, metabolizable energy (ME) value (MJ), and essential nutrients of experimental diets (g/kg of feed).

Ingredients	Starter (1–10 d)				Grower (11–25 d)				Finisher (26–43 d)			
	CT	EX5	EX10	EX15	CT	EX5	EX10	EX15	CT	EX5	EX10	EX15
Ground maize	403.0	432.9	459.9	486.7	443.7	470.6	496.5	523.4	446.5	472.4	499.3	526.2
Ground wheat	150	100	50	0	150	100	50	0	150	100	50	0
Soybean meal	358	326	296	266	304	274	245	215	303	274	244	214
Rapeseed oil	48	50	53	56	60	63	66	69	65	68	71	74
Wheat germ expeller	0	50	100	150	0	50	100	150	0	50	100	150
Sodium bicarbonate	5.40	5.40	5.32	5.32	5.77	5.74	5.72	5.69	5.77	5.75	5.72	5.68
Monocalcium phosphate	13.7	13.7	13.6	13.6	12.9	12.8	12.8	12.7	12.0	12.0	11.9	11.9
Limestone	13.7	13.8	14.0	14.2	14.1	14.3	14.5	14.7	12.6	12.9	13.1	13.3
L-Lysine	2.14	2.21	2.24	2.27	2.86	2.89	2.89	2.92	0.84	0.86	0.87	0.91
DL-Methionine	2.77	2.61	2.45	2.29	2.73	2.57	2.40	2.25	1.66	1.50	1.33	1.17
L-Threonine	0.71	0.87	1.01	1.15	1.49	1.62	1.75	1.90	0.07	0.21	0.34	0.48
Premix 0.25%	2.50	2.50	2.50	2.50	2.50	2.50	2.50	2.50	2.50	2.50	2.50	2.50
ME [MJ/kg of feed] value and essential nutrients of experimental diets [g/kg of feed]												
ME	12.50	12.49	12.50	12.50	12.98	12.99	12.99	12.99	13.19	13.19	13.19	13.19
Dry matter	892	899	905	910	893	901	907	912	893	901	906	911
Crude protein	221	220	220	220	200	200	200	200	200	200	200	200
Crude fiber	28.3	27.2	26.2	25.1	27.1	26.1	25.0	24.0	27.1	26.1	25.0	24.0
Ca	9.45	9.40	9.40	9.40	9.20	9.21	9.20	9.20	8.50	8.51	8.50	8.50
P available	4.30	4.30	4.30	4.30	4.00	4.00	4.00	4.00	3.80	3.80	3.80	3.80
Na	1.60	1.61	1.60	1.60	1.70	1.70	1.70	1.70	1.70	1.70	1.70	1.70
Lysine (total)	12.00	12.00	12.00	12.00	11.51	11.51	11.50	11.50	9.50	9.52	9.50	9.51
Methionine (total)	5.50	5.50	5.50	5.50	5.20	5.20	5.20	5.21	4.30	4.30	4.30	4.30
Threonine (total)	8.01	8.00	8.00	8.00	8.01	8.00	8.00	8.01	6.60	6.61	6.60	6.60

Abbreviations: CT, Control Treatment; EX5, Experimental diet with 5% wheat germ expeller; EX10, Experimental diet with 10% wheat germ expeller; EX15, Experimental diet with 15% wheat germ expeller. The content of additives added in the premix (0.25%) in 1 kg of the mixture: Vitamin A—8100 UI.; D3—3000 UI.; E—22.50 UI.; K3—1.25 mg; B1—1.25 mg. B2—4.60 mg; B6—2.20 mg; B12—0.02 mg; PP—20.0 mg; choline chloride—184.25 mg calcium D-pantothenate—6.50 mg; folic acid—0.34 mg; biotin—0.10 mg; betaine; hydrochloride—41.89 mg; Cu—10 mg; Fe—30 mg; Zn—60 mg; Mn—70.40 mg; J—0.75 mg; Se—0.20 mg; Substances improving digestibility: Endo-1.4.beta-xyl—300 U; Subtilisin—4.000 U; Alpha-amyl—400 U; Endo-1.4-beta-xylanase—1.525 U. Endo-1.3(4)-beta-glucanase—190 U; 6-phytase—500 FTU.

Table S2. Chemical composition of wheat germ expeller (Mean, SD).

Items	Unit	Wheat Germ Expeller
Gross energy	MJ kg^{-1} of feed	14.14
Crude protein	%	35.6 ± 2.5
Crude fat	%	6.0 ± 0.5
Carbohydrates	%	27.8
Crude ash	%	4.75 ± 0.20
Water and volatiles	%	11.6 ± 0.8
Total fiber	%	2.8 ± 0.3

Table S3. Chemical composition of the experimental diets (applies to the finisher's diet).

Items	Unit	CT [1]	EX5 [2]	EX10 [3]	EX15 [4]
Gross energy	MJ kg^{-1} DM	19.71	19.80	19.70	19.82
Dry matter	%	90.59	90.66	91.51	91.71
Crude protein	%	23.38	23.46	23.14	22.98
Ether extract	%	6.06	6.09	6.67	7.15
Crude ash	%	5.31	4.84	5.91	5.72
Nitrogen free extractives	%	52.77	53.13	52.58	53.28
Crude fiber	%	3.07	3.14	3.21	2.63
Crude Protein: Energy ratio		1.19	1.18	1.17	1.16
	mg kg^{-1} DM	Macroelements			
Pabs (absorbable phosphorus)		5980.0	6090.0	7010.0	6970.0
Na		1199.4	1161.2	1170.2	1175.5
Ca		7582.3	8537.6	10385.5	9086.2
K		488.0	477.0	435.0	447.0
Mg		161.0	181.0	174.0	177.0
		Microelements			
Fe		173.4	165.1	180.7	184.3
Zn		74.2	73.6	80.5	78.1
Cu		14.4	11.3	11.8	11.8
Mn		78.9	86.4	90.1	94.9

[1] CT, Control Treatment; [2] EX5, Experimental diet with 5% wheat germ expeller; [3] EX10, Experimental diet with 10% wheat germ expeller; [4] EX15, Experimental diet with 15% wheat germ expeller.

Table S4. Feed intake and weight gain of male 43-day-old Ross-308 broilers (MEAN, SEM, $n = 32$).

Items	CT [1]	EX5 [2]	EX10 [3]	EX15 [4]	SEM [5]	*p* Value [6]
Final body live weight (g)	2514.4 [a]	2379.4	2270.6 [b]	2264.4 [b]	35.8	0.042
Feed consumption (g per 100 g body weight^{-1})	169.7 [B]	194.1 [A]	197.1 [A]	193.9 [A]	0.004	<0.001
Body weight gain (g per 100 g feed^{-1})	58.2 [A]	51.2 [B]	50.1 [B]	50.8 [B]	0.854	<0.001
Gross energy intake (kJ per 100 g body weight^{-1})	32.5 [B,b]	36.3 [B,a]	41.2 [A]	34.7 [B]	0.720	<0.001
Protein intake (g per 100 g body weight^{-1})	39.7 [B,b]	45.5 [A]	45.6 [A]	44.6 [a]	0.686	<0.001
Fat intake (g per 100 g body weight^{-1})	10.3 [B]	11.8 [A,D,b]	13.1 [A,a]	13.9 [A,C]	0.284	<0.001
Protein efficiency ratio (PER)	2.49 [A,a]	2.18 [B]	2.16 [B]	2.21 [b]	0.036	<0.001
Feed conversion efficiency (FCE)	0.58 [A]	0.51 [B]	0.50 [B]	0.51 [B]	0.008	<0.001
Feed conversion ratio (FCR)	1.70 [B]	1.94 [A]	1.97 [A]	1.94 [A]	0.030	<0.001
Macroelements consumption (mg per 100 g body weight^{-1})						
P	1014.7 [B,D]	1181.9 [B,C]	1381.9 [A]	1351.4 [A]	30.1	<0.001
Na	2035.2 [B,b]	2253.6 [a]	2306.9 [A]	2279.1 [a]	32.7	0.005
Ca	12899.0 [B,D]	16568.9 [B,C]	20473.9 [A]	17616.8 [A]	529.4	<0.001
K	828.1 [b]	925.7 [a]	857.6	866.7	12.3	0.038
Mg	273.2 [B]	351.3 [A]	343.0 [A]	343.2 [A]	6.95	<0.001
Microelements consumption (mg per 100 g body weight^{-1})						
Fe	294.2 [B]	320.4 [b]	356.2 [A,a]	357.4 [A,a]	8.13	<0.001
Zn	125.8 [B]	142.8 [A,b]	158.8 [A,a]	151.3 [A]	2.79	<0.001
Cu	24.3	23.8	23.2	22.8	0.29	0.316
Mn	133.8 [B]	168.1 [A]	177.6 [A]	183.9 [A]	3.99	<0.001

[1] CT, Control Treatment; [2] EX5, Experimental diet with 5% wheat germ expeller; [3] EX10, Experimental diet with 10% wheat germ expeller; [4] EX15, Experimental diet with 15% wheat germ expeller; [5] SEM, standard error of the mean; BW: body weight; [6] Means within a row followed by different superscript letters differ significantly [A,B,C,D] $p \leq 0.01$; [a,b] $p \leq 0.05$.
